# *Ceratonia siliqua* L. Pod Extract: From Phytochemical Characterization to Liposomal Formulation and Evaluation of Behaviour in Cells

**DOI:** 10.3390/antiox12061209

**Published:** 2023-06-03

**Authors:** Maria De Luca, Carlo Ignazio Giovanni Tuberoso, Ramon Pons, María Teresa García, María del Carmen Morán, Giuseppe Martelli, Antonio Vassallo, Carla Caddeo

**Affiliations:** 1 Department of Science, University of Basilicata, Viale dell’Ateneo Lucano 10, 85100 Potenza, Italy; maria.deluca@unibas.it (M.D.L.); giuseppe.martelli@unibas.it (G.M.); 2 KAMABIO Srl, Via Al Boschetto 4/B, 39100 Bolzano, Italy; 3 Department of Life and Environmental Sciences, University of Cagliari, S.P. Monserrato-Sestu km 0.700, 09042 Monserrato, Italy; tuberoso@unica.it (C.I.G.T.); caddeoc@unica.it (C.C.); 4 Department of Surfactants and Nanobiotechnology, Institute for Advanced Chemistry of Catalonia (IQAC-CSIC), c/Jordi Girona 18–26, 08034 Barcelona, Spain; ramon.pons@iqac.csic.es (R.P.); teresa.garcia@iqac.csic.es (M.T.G.); 5 Department of Biochemistry and Physiology, Physiology Section, Faculty of Pharmacy and Food Science, University of Barcelona, Avda. Joan XXIII 27–31, 08028 Barcelona, Spain; mcmoranb@ub.edu; 6 Institute of Nanoscience and Nanotechnology-IN2UB, University of Barcelona, Avda. Diagonal 645, 08028 Barcelona, Spain; 7 Spinoff TNcKILLERS Srl, Viale dell’Ateneo Lucano 10, 85100 Potenza, Italy

**Keywords:** *Ceratonia siliqua*, extract, liposomes, biocompatibility, antioxidant, erythrocytes, skin cells

## Abstract

The formulation of plant extracts in phospholipid vesicles is a promising strategy to exploit their biological properties while solving problems related to poor solubility in water, high instability, and low skin permeation and retention time. In this study, *Ceratonia siliqua* ripe pods were used for the preparation of a hydro-ethanolic extract, which showed antioxidant properties owing to the presence of biologically active compounds identified by liquid chromatography–mass spectrometry (e.g., hydroxybenzoic acid and flavonoid derivatives). To improve the applicability of the extract in therapy, a topical formulation based on liposomes was explored. The vesicles were characterized by small size (around 100 nm), negative charge (−13 mV), and high entrapment efficiency (>90%). Furthermore, they displayed both spherical and elongated shapes, with oligolamellar structure. Their biocompatibility was demonstrated in cells, including erythrocytes and representative skin cell lines. The antioxidant activity of the extract was proved by the scavenging of free radicals, the reduction of ferric ions, and the protection of skin cells from oxidative damage.

## 1. Introduction

*Ceratonia siliqua* L., commonly called carob, is an evergreen tree that belongs to the Leguminosae family widely cultivated in Mediterranean countries [1]. Traditionally, carob has been used to produce animal feed. Nowadays, agricultural and industrial sectors exploit carob fruit and its primary products (i.e., flour, powder, and syrup) to develop a variety of foods and beverages [2]. The fruit is a brown pod with an elongated and compressed shape of varying dimensions and a wrinkled surface that becomes leathery when ripe. The pods are mainly made up of sweet edible pulp with a leathery outer layer (pericarp) and a softer inner area (mesocarp), rich in hard seeds [3]. Carob pulp contains a wide range of biologically active compounds [4]. Generally, carob pods have a high sugar content, relatively low content of lipids and protein, and some essential amino acids (aspartic and glutamic acids), as well as ω-3 and ω-6 fatty acids (oleic, linoleic, and α-linolenic acids). Moreover, the fruit contains a high amount of low-calorie dietary fibers (cellulose, hemicelluloses, and lignin), minerals (calcium, phosphorus, and potassium), and phenolic compounds [2,5]. The phenolic content is mainly represented by gallic acid; the other phenolic compounds are myricetin rhamnocyte, quercetin rhamnocyte, methyl gallate, cinnamic acid, and myricetin glycoside [3,6,7]. Carob pods show significant pharmacological activities (anti-inflammatory, antibacterial, antidiabetic, antihypercholesterolemic, hepatoprotective, neuroprotective, and nephroprotective) [1,4,8,9,10]. Traditional medicine used carob pods for the treatment of human gastrointestinal diseases. Several studies showed that carob pods could be useful for the attenuation of processes related to chronic diseases, such as type 2 diabetes, obesity, and metabolic syndrome [11]. They exert beneficial effects on dyslipidemia and interfere with glucose absorption mechanisms [12,13,14]. Many of these activities are related to the inhibiting potential of oxidant species [15].

In this study, the antioxidant activity of a hydro-alcoholic extract obtained from ripe carob pods was studied with the aim of a possible utilization on the skin. As is known, free radicals significantly contribute to skin damage and accelerate ageing by disrupting the body’s defenses and restoration mechanisms [16]. The topical application of natural compounds with antioxidant activity is often limited by poor aqueous solubility, high chemical instability, and low skin permeation. The nanoformulation of plant extracts in phospholipid vesicles is a promising strategy to overcome these drawbacks and exploit their biological properties. Therefore, the antioxidant properties of the *C. siliqua* hydro-alcoholic extract, both free in solution and formulated in liposomes, were investigated. The antioxidant studies were performed in vitro by spectrophotometric assays (DPPH and FRAP), and by assessing the prevention of hydrogen-peroxide-induced oxidative damage in fibroblasts and keratinocytes. In addition, the biocompatibility of the nanoformulations was evaluated in cell models (i.e., erythrocytes and skin cells).

## 2. Materials and Methods

### 2.1. Reagents and Standards

Ethanol absolute was from VWR (Milan, Italy); methanol and 85% *w*/*w* phosphoric acid were from Sigma-Aldrich (Steinheim, Germany); LC–MS-grade acetonitrile, formic acid, and water were from Merck (Darmstadt, Germany). Standards of gallic acid, methyl gallate, ethyl gallate, myricetin 3-*O*-glucoside, quercetin, quercetin-3-*O*-rhamnoside, and kaempferol-3-*O*-glucoside were from Extrasynthese (Genay, France) and TransMIT (Giessen, Germany). Phospholipon90G (>94% phosphatidylcholine; P90G) was from Lipoid GmbH (Ludwigshafen, Germany). All the reagents for cell culture were provided by Lonza (Verviers, Belgium).

### 2.2. Extract Preparation

*Ceratonia siliqua* L. ripe pods were collected in the Arco Ionico Metapontino area (Basilicata, Italy) in 2019. The pods were cut into small pieces and left to dry for 72 h. Subsequently, the samples were ground in a food processor to produce a fine powder and sieved through a stainless-steel mesh. The powder was dispersed in a 70:30 *v*/*v* ethanol: water mixture (powder: solvent ratio 1:2 *w*/*v*), sonicated for 30 min at room temperature, and macerated for 24 h. The macerate was filtered and another aliquot of the ethanol: water mixture was added. The procedure was repeated twice. The extractive solutions were filtered again and concentrated using a rotary evaporator. The obtained extract was stored at −20 °C until use.

### 2.3. High-Resolution HPLC-ESI-QToF-MS/MS and HPLC-PDA Analysis

The qualitative investigation of the *C. siliqua* pod extract was performed by an ion mobility QToF LC/MS system according to De Luca et al. [17], using a 1290 Infinity II UPLC equipped with a 6560 IM-QToF (Agilent Technologies Inc., Palo Alto, CA, USA). Data acquisition and processing were performed using the Agilent MassHunter Workstation Acquisition software v. B.09.00 (Agilent Technologies). ESI/QToF MS data were then analyzed using the MassHunter Workstation Qualitative Analysis software v. 10.0 (Agilent Technologies), and the MassHunter METLIN metabolite PCDL database v. B.08.00 (Agilent Technologies) and the Sirius^®^ software v. 4.7.4 were used for the tentative identification of the metabolites and to predict fragmentation and molecular formulae [18,19].

The quantitative analysis of targeted phenolic compounds was performed by using an HPLC-photodiode array (PDA) detection method reported by De Luca et al. [20] with an Agilent 1260 Infinity II HPLC system and an Agilent G4212B photodiode array detector (Agilent Technologies). The chromatograms and spectra were elaborated with an OpenLab v. 2.51 data system (Agilent Technologies) and phenolic compounds were detected and quantified at the following wavelengths: flavonoids at 360 nm and hydroxybenzoic acids at 280 nm. For the quantitative analysis, the extract was dissolved in an 80:20 *v*/*v* MeOH:H_2_O mixture (extract–solvent ratio 1:50 *w*/*v*) and diluted 1:1 *v*/*v* with 0.22 M H_3_PO_4_. The extract liposomes were injected after dilution (1:100 *v*/*v*) with MeOH and filtration with 0.22 μm CA syringe filters. Phenolic compounds amount was expressed as mg/g dr (dried extract).

### 2.4. Preparation of Liposomes

The *C. siliqua* extract was formulated in liposomes, which were prepared by sonicating P90G and the extract dispersed in water (13 cycles of 5 s on/2 s off + 5 cycles 2 s on/2 s off; 13 µm of probe amplitude). To allow proper comparisons, empty liposomes were prepared according to the procedure used for extract-loaded liposomes, but without the extract (Table 1).

### 2.5. Characterization of Liposomes

The average diameter, polydispersity index, and zeta potential of the liposomes were measured with a Zetasizer nano-ZS (Malvern Panalytical, Worcestershire, UK) through dynamic and electrophoretic light-scattering. The liposome dispersions were diluted (1:100 *v*/*v*) with water and analyzed at 25 °C.

The liposomes were observed by cryogenic transmission electron microscopy (cryo-TEM) using a JEM-2011 transmission electron microscope (JEOL USA Inc., Peabody, MA, USA) at an accelerating voltage of 200 kV. The liposomes (4 μL) were applied onto a holey carbon grid, which was plunge-frozen into liquid ethane (−180 °C) after removing excess fluid by automatic blotting using a Leica EM GP cryo-preparation chamber (Leica Microsystems Inc., Deerfield, IL, USA). The sample was vitrified to prevent radiation damage and preserve the vesicle structure.

To calculate the entrapment efficiency (EE) of the liposomes, a dialysis against water was performed to remove the extract components nonincorporated into the vesicles. The liposomes (1 mL) were loaded into Spectra/Por^®^ membranes (12–14 KDa MWCO; Spectrum, Breda, The Netherlands) and dialyzed against water for 2 h. Nondialyzed and dialyzed liposomes were diluted (1:100 *v*/*v*) with methanol and analyzed by HPLC-PDA to quantify marker extract compounds, according to the procedure described in Section 2.3 and applying the following Formula (1):

(1) $$EE=\left(\frac{quantity\;of\;compound\;indialyzed\;vesicles}{quantity\;of\;compound\;in\;non-dialyzed\;vesicles}\right)\times 100$$

### 2.6. Small-Angle X-ray Scattering

A deep characterization of the liposomes bilayer was performed by small-angle X-ray scattering (SAXS) analyses by using an in-house instrument. The details of the equipment and the experimental conditions can be found in De Luca et al. [17]. The scattering curves were recorded every 20 min up to 2 h to check for sample stability; those curves were summed up and background was subtracted. SAXS patterns were analyzed using a homemade fitting procedure based on a Gaussian description of the bilayers and using a Levenberg–Marquardt minimization scheme [21,22,23,24,25], which takes into account the instrumental convolution for detector width and beam profile.

### 2.7. Antioxidant Activity: DPPH and FRAP Assays

The DPPH assay allows the determination of the antioxidant power of a sample by monitoring the reduction reaction of the DPPH free radical (1,1-diphenyl-2-picrylhydrazyl). The unpaired electron of the DPPH radical absorbs at 517 nm and exhibits an intense purple color in solution. The radical is neutralized by accepting either a hydrogen atom or an electron from an antioxidant species with a concomitant discoloration to pale yellow. The decrease in absorbance is proportional to the antioxidant power of the sample.

Ten µL of each sample were added to a 25 µM DPPH methanolic solution and incubated at room temperature in the dark for 30 min. The color change of the solution was detected through light adsorbed at 517 nm. The antioxidant activity (AA) of the extract samples was calculated according to the following Formula (2):

(2) $$AA(\%)=\left(\frac{A_{DPPH}-A_{sample}}{A_{DPPH}}\right)\times 100$$

The results were expressed also as Trolox equivalents (µg TE/mL solution) calculated by using a calibration curve (Trolox concentration range: 0–500 µg/mL).

The ferric ion reducing antioxidant power, or FRAP, is based on the reduction of the Fe^3+^-TPTZ (iron-2,4,6-tripyridyl-S-triazine) complex, under acidic conditions, to the intense blue-colored ferrous complex Fe^2+^-TPTZ, which causes an increase in absorbance. FRAP reagent was prepared by mixing 0.3 mM TPTZ and 20 mM FeCl_3_ × 6H_2_O in 0.2 M acetate buffer (pH 3.6).

The sample (10 μL) was added to a 2 mL FRAP reagent and incubated at room temperature for 4 min in the dark; the absorbance was read at 593 nm. The results, expressed as µg Fe^2+^ equivalents/mL solution, were calculated by using a calibration curve (FeSO_4_ concentration range: 13.9–2502 µg/mL).

### 2.8. Liposomes’ Biocompatibility: Hemolytic Activity and Cell Viability

The biocompatibility was assayed through the hemolytic activity evaluation according to a procedure described in the literature [26]. The *C. siliqua* extract samples were dissolved in a total volume of 1 mL with phosphate buffered saline (PBS; pH 7.4) and 25 μL of an erythrocyte suspension. Erythrocytes were isolated from rabbit blood samples supplied by the animal facility of the Research and Development Center (CID)—Spanish National Research Council (CID-CSIC, Barcelona, Spain). The blood samples were collected in strict compliance with the bioethical principles established by the Spanish legislation. The study was approved by the Animal Experimentation Ethics Committee of the Research and Development Center (CEEA-CID, CSIC). The erythrocytes were washed three times in PBS and resuspended in PBS at a cell density of approximately 10^9^ cells/mL. The assay was performed using 50 and 100 µL of an extract solution (20 mg/mL in 70:30 *v*/*v* ethanol:water) or liposomes (20 mg/mL), and 0% and 100% hemolysis controls (erythrocytes in PBS and in ultrapure water, respectively). Empty liposomes were also assayed for a proper comparison. The samples were incubated for 10 min at room temperature, under stirring, and then centrifuged (5 min at 10,000 rpm). Hemolysis (%) was calculated as a function of the absorbance at 575 nm of the supernatant of the *C. siliqua* samples in comparison with that of the controls.

The biocompatibility of *C. siliqua* samples was also tested in three skin cell lines. Murine Swiss albino fibroblasts (3T3), immortal human keratinocytes (HaCaT), and squamous carcinoma cells (A431) were provided by Celltec UB (Barcelona, Spain). The cells were grown in Dulbecco’s Modified Eagle’s Medium (DMEM) high glucose (4.5 g/L) with 10% (*v*/*v*) fetal bovine serum (FBS), 2 mM *L*-glutamine, 100 U/mL penicillin, and 100 μg/mL streptomycin under standard conditions (37 °C, 5% CO_2_). The cells were trypsinized when approximately 80% confluent and seeded into 96-well plates (3T3 and HaCaT cells at 1 × 10^5^ cells/mL, A431 cells at 5 × 10^4^ cells/mL). After 24 h, the medium was removed, and the cells were incubated for another 24 h with *C. siliqua* extract in solution (70:30 *v*/*v* ethanol:water) or in liposomes, previously diluted with the culture medium to achieve the required concentrations (1–200 μg/mL). Empty liposomes were also tested for a proper comparison. After the incubation time, the medium was removed and cell viability was tested by the MTT assay, which relies on the ability of living cells to convert yellow MTT (2,5-Diphenyl-3-(4,5-dimethyl-2-thiazolyl) tetrazolium bromide) into purple formazan. A total of 100 μL of MTT (5 mg/mL in PBS then diluted (1:10 *v*/*v*) with DMEM without phenol red nor FBS) was added to the cells. After 3 h, the MTT was removed and 100 μL of dimethylsulfoxide was added to dissolve the formazan crystals. The absorbance of the solutions was read at 550 nm using a Bio-Rad 550 microplate reader (Hercules, CA, US). The cell viability results were expressed as the percentage of the MTT reduction in treated cells with respect to untreated control cells (100% viability).

### 2.9. Antioxidant Activity in Cell Lines

The protective capacity of *C. siliqua* extract, free in solution or formulated in liposomes, against hydrogen-peroxide-induced oxidative stress was evaluated in cells. A 2 mM concentration of H_2_O_2_ was chosen after assaying the induced cytotoxicity in three cell lines (i.e., 3T3, HaCaT, A431 cells). After 24 h of incubation of the cells with the samples under investigation (100 µg/mL of extract in solution or in liposomes, and empty liposomes), the medium was removed, and the H_2_O_2_ was added. After 3 h of incubation, the MTT assay was performed under the conditions described in Section 2.8. The antioxidant activity of the samples was expressed as the percentage of the protective capacity (PC) against the cytotoxicity induced by H_2_O_2_ according to the following Formula (3):

(3) $$PC(\%)=\frac{Cell\;viability\;induced\;by\;samples-Cell\;viability\;induced\;by\;H_{2}O_{2}}{Cell\;viability\;induced\;by\;samples}\times 100$$

### 2.10. Statistical Analysis

Results are reported as means ± standard deviation (SD). Student’s *t*-test was used to determine the significant differences between groups. For cells experiments, results are reported as means ± standard error (SE). One-way ANOVA was used to determine differences between datasets, and the Scheffé post hoc test was used for multiple comparisons. *p* values < 0.05 were considered statistically significant.

## 3. Results

### 3.1. Quali–Quantitative Phenolic Profile of C. siliqua Pod Extract

The *C. siliqua* pod extract was qualitatively analyzed by HPLC-ESI-QToF MS/MS in negative ion mode, and phenolic compounds were quantified by HPLC-PDA.

**Figure 1:**
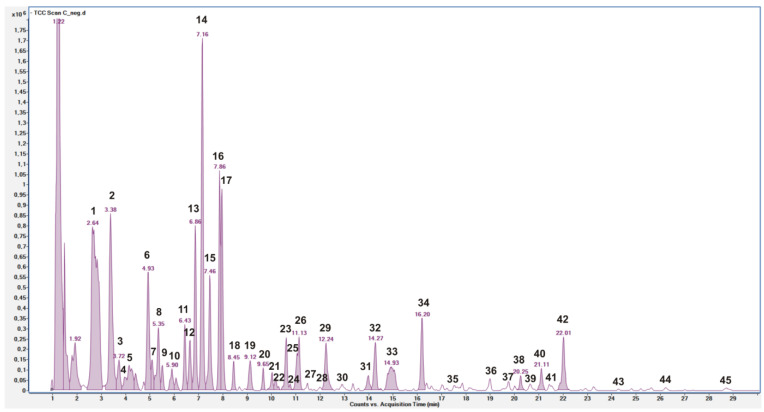
HPLC-ESI-QToF MS total compound chromatogram of *C. siliqua* pod extract acquired in negative ion mode. Peaks identification is reported in Table 2.

The LC–MS profile (Figure 1) displayed a large number of compounds, which were identified by comparison of their *m*/*z* values in the total compound chromatogram profile with those described in the literature, and of the experimental MS/MS spectra with fragmentation patterns reported in the literature or with spectra reported in a public repository [18,19,27].

Table 2 reports the compounds identified by MS data, listed according to their retention times, chemical formula derived by accurate mass measurement, MS/MS results, references used for identification, and the identification confidence levels [28]. Forty compounds were tentatively identified as sugars, hydroxybenzoic acid, and flavonoid derivatives, and other five remained unknown.

Fourteen peaks were tentatively identified as sugars derivatives, namely, acylated disaccharide [5,29]. Peaks **10**, **16**, **17**, and **18**, with [M-H]^−^ at *m*/*z* 411.1518, corresponding to a molecular formula of C_16_H_28_O_12_, were attributed to (iso)butyryl-dihexose isomers [29]. Other isomers of the same compound were also detected as formic acid adduct (peaks **3** and **15**). Peaks **11**, **13**, and **14**, with [M-H]^−^ at *m*/*z* 427.1466, were attributed to the formic acid adduct of (iso)butyryl-hexose-pentose corresponding to a molecular formula of C_15_H_26_O_11_ [29]. Peaks **22** and **23**, with [M-H]^−^ at *m*/*z* 441.1615, were attributed to the formic acid adduct of acilated-hexose-pentose, corresponding to a molecular formula of C_16_H_28_O_11_ [29]. Peaks **27** and **28**, with [M-H]^−^ at *m*/*z* 461.1296, were attributed to the formic acid adduct of acilated-hexose-pentose corresponding to a molecular formula of C_18_H_24_O_11_ [29].

Nineteen compounds were identified as hydroxybenzoic acid derivatives, mainly galloyl glucose derivatives (gallotannins), due to the typical fragment ion at *m/z* 169, which was associated with the gallic acid unit [5,29]. Peak **2**, with [M−H]^−^ at *m/z* 169.01476, was absolutely identified as gallic acid by comparing with its commercial standard. Peaks **1** and **4**, with [M−H]^−^ at *m*/*z* 331, were attributed to gallic acid glucosides [5,29]. Peaks **5**, **12**, and **24**, with [M-H]^−^ at *m*/*z* 483.0783, were attributed to different digalloyl-glucose isomers, and peaks **26**, **30**, and **33**, with [M-H]^−^ at *m*/*z* 635.0888, to trigalloyl-glucose isomers. No traces of tetra-galloyl-glucose were detected. Peaks **6**, **8**, and **9**, with [M-H]^−^ at *m/z* 493.12008 corresponding to a molecular formula of C_19_H_26_O_15_, were attributed to monogalloyl dihexoside isomers [5,30]. Peak **19**, with [M-H]^−^ at *m*/*z* 183.0300, was attributed to a methyl gallic acid derivative. Farag et al. [5] attributed a compound with similar characteristics to methyl gallate, but a comparison with the pure commercial standard excluded this attribution. Peak **32**, with [M-H]^−^ at *m/z* 197.0460, was consistent with ethyl gallate [32], but a comparison with the pure commercial standard excluded this attribution and the peak was attributed to a ethyl gallic acid derivative. Peak **29**, with [M-H]^−^ at *m*/*z* 595.1309, was attributed to a digalloyl derivative [5], tentatively siliquapyranone [29]. Peak **34**, with [M-H]^−^ at *m*/*z* 401.1095, and peak **40**, with [M-H]^−^ at *m*/*z* 507.11438, were attributed to gallic acid derivatives [5]. Peak **35**, with [M-H]^−^ at *m*/*z* 121.02935, was attributed to benzoic acid [31]. Hydroxybenzoic acids were the most abundant compounds in the extract, accounting for 7.19 ± 0.37 mg/g dr. Compounds **1**, **2**, and **34** were the most abundant, and the sum of monogalloyl dihexosides (0.81 ± 0.04 mg/g dr) was higher than both sums of digalloyl glucose and trigalloyl glucose isomer (0.43 ± 0.02, and 0.44 ± 0.03 mg/g dr, respectively).

Seven peaks were attributed to flavonoid derivatives. Peaks **36** and **37**, with [M-H]^−^ at *m/z* 463.0876, were attributed to flavonol monoglycoside, and from fragment patterns they were tentatively attributed to myricitrin deoxyhexoside and quercetin hexoside [5,29]. Peak **39**, with [M-H]^−^ at *m*/*z* 433.07735, was attributed to a quercetin pentoside [5,29]. Peak **42**, with [M-H]^−^ at *m*/*z* 447.09389, was attributed to quercetin deoxyhexoside [5,29], and to quercetin-3-*O*-rhamnoside by comparison with its commercial standard. Peak **43**, with [M-H]^−^ at *m*/*z* 431.0988, was attributed to kaempferol deoxyhexose. Peak **44**, with [M-H]^−^ at *m*/*z* 287.05581, was attributed to a tetrahydroxy flavanone [5]. Peak **45**, with [M-H]^−^ at *m*/*z* 285.0441, was attributed to kaempferol by comparison with its commercial standard [5]. From a quantitative point of view, the total flavonoid amount was 0.37 ± 0.02 mg/g dr, with quercetin-3-*O*-rhamnoside accounting for more than 58% (0.21 ± 0.00 mg/g dr; Table 3).

### 3.2. Liposomes’ Characteristics

Liposomes loaded with *C. siliqua* extract were approximately of 100 nm and significantly larger in mean diameter than empty liposomes (73 nm; Table 4). The polydispersity index and zeta potential values for *C. siliqua* liposomes were 0.27 and −13 mV, respectively, similar to those measured for the empty liposomes (Table 4).

**Figure 2:**
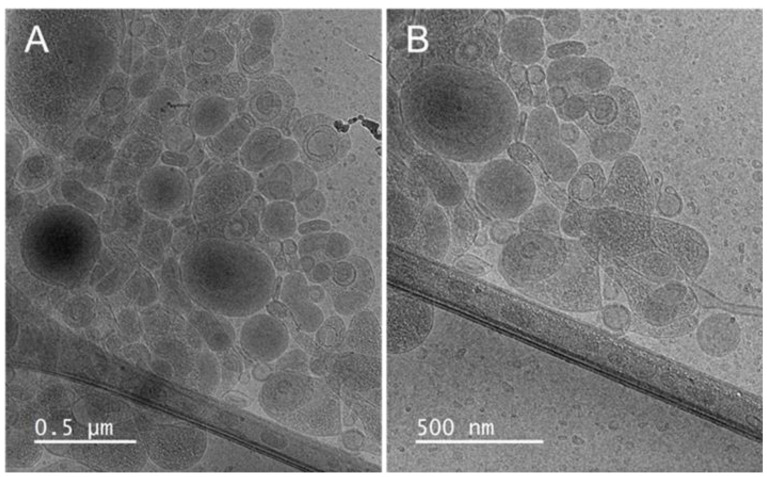
Cryo-TEM images of *C. siliqua* liposomes. Two magnifications are shown: 12,000× (**A**) and 15,000× (**B**).

The formation of vesicular structures was confirmed by cryo-TEM analysis. Figure 2 shows the presence of both spherical and elongated oligolamellar vesicles at around 100 nm in diameter, which aligns with the light scattering results (Table 4).

**Figure 3:**
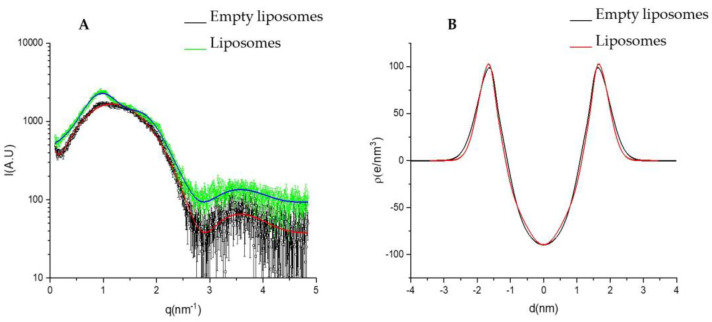
(**A**) SAXS patterns of empty liposomes and *C. siliqua* extract liposomes. The best fits of bilayer Gaussian description of the electronic profiles are shown as lines. (**B**) Electron density profiles used for the fitting of empty liposomes and *C. siliqua* extract liposomes.

A deeper structural characterization of liposomes was gained by SAXS analysis. The SAXS patterns of liposomes are shown in Figure 3, together with the fits of the lamellar model (*χ^2^* = 1.75 and 1.61), which were typical of bilayered structures. The main parameters obtained from the fits are listed in Table 5. The results suggest that the *C. siliqua* extract induced in liposomes some multilamellar arrangement with a small number of correlated layers (*N* = 1.26) at a repetition distance *d* of ~62 Å, and a Caillé parameter *η*_1_ = 0.23, which is indicative of flexible bilayers. The distance between the polar heads and the bilayer center (*Z_H_*) slightly increased with the extract’s loading. The polar head (*σ_H_*) and methyl (*σ_C_*) amplitude slightly decreased in liposomes loaded with the extract; however, the differences were small and just above the limit to be considered significant for *σ_H_* and *Z_H_*. Therefore, the presence of the extract affected the bilayered structure moderately.

The entrapment efficiency of *C. siliqua* extract in liposomes was assayed through HPLC quantification of two targeted phenolic compounds (Table 6). The liposomes were capable of entrapping a high amount of extract, since the entrapment efficiency was 97% ± 6.3 for the gallic acid derivative and 91% ± 7.7 for the quercetin-3-*O*-rhamnoside.

### 3.3. Liposomes’ Biocompatibility

The biocompatibility of the investigated samples was first assayed on erythrocytes as hemolytic activity evaluation. The results are shown in Table 7. All the samples showed a negligible erythrocyte-disrupting ability. More precisely, the hemolytic activity was lower than 5%, without statistically significant differences between the free and the nanoformulated forms of the extract.

The treatment of three skin cell lines with the extract, free in solution or nanoformulated in liposomes, at the tested concentrations, was not toxic, as expressed by the MTT results.

**Figure 4:**
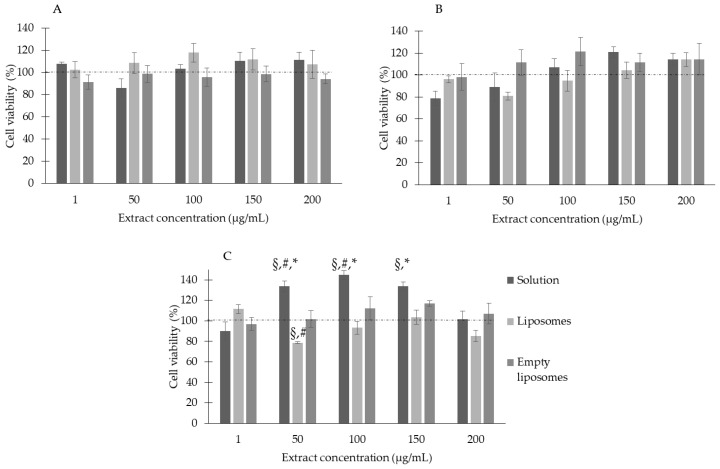
Viability of 3T3 (**A**), HaCaT (**B**), and A431 (**C**) cells upon exposure to *C. siliqua* samples for 24 h. Data are expressed as means ± standard error (SE), *n* = 6. ^§^ *p* < 0.05 vs. untreated control cells (100% viability); * *p* < 0.05 vs. liposomes; # *p* < 0.05 vs. empty liposomes.

For 3T3 fibroblasts, the MTT results showed that cell viability values were never lower than 86%. After treatment with the extract in the liposomal form, the cell viability values were approximately the same as for the untreated control cells. Similarly, empty liposomes did not show cytotoxicity, and no statistically relevant difference was highlighted among the different groups (Figure 4A). In the case of normal HaCaT keratinocytes, the same results were obtained: the cells showed viability values always higher than 80% when treated with the extract solution or liposomes, both with the same trend of proliferation at increasing concentrations, but without statistically relevant differences. For the tumoral A431 keratinocytes, there was a statistically relevant difference between the extract solution and the liposomes; particularly, cell viability was slightly affected by liposomal treatment. Nevertheless, the lower value was approximately 80% (Figure 4C). In all cases, cells treated with empty liposomes exhibited no cytotoxicity, confirming the biocompatibility of the nanocarriers.

### 3.4. Antioxidant Activity

The antioxidant activity of the *C. siliqua* extract was determined as a function of its radical scavenging and ferric reducing abilities. The extract solution scavenged the DPPH radical almost completely (92%, corresponding to 469 μg/mL of Trolox equivalents; Table 8). The level of antioxidant activity for the extract liposomes was slightly higher (95%, corresponding to 486 μg/mL of Trolox equivalents; Table 8). Given the presence of phosphatidylcholine, empty liposomes possessed a moderate antioxidant activity (40%; Table 8).

The results of the FRAP assay showed that both the extract solution and the extract liposomes possessed a strong reducing power (~2000 μg/mL of ferrous equivalents; Table 9), without statistically relevant differences. The empty liposomes showed minimal activity (Table 9). These findings demonstrate that the strong antioxidant activity of the *C. siliqua* extract was retained in the liposome formulation.

**Figure 5:**
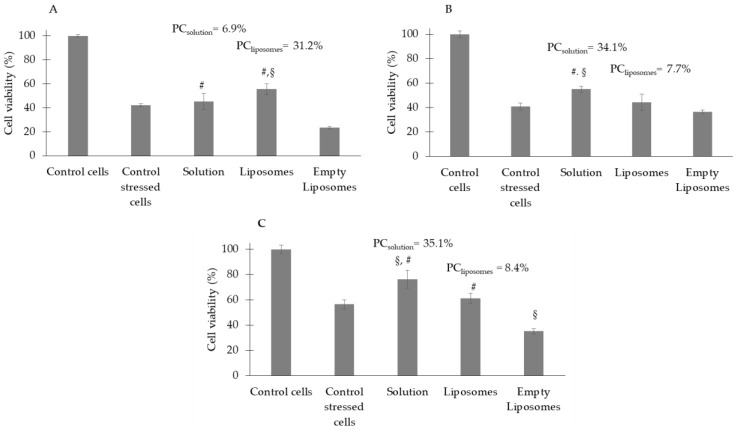
Viability of 3T3 (**A**), HaCaT (**B**), and A431 (**C**) cells upon pretreatment with *C. siliqua* samples and subsequent exposure to 2 mM H_2_O_2_. Data are expressed as means ± standard error (SE); *n* = 3; ^§^ *p* < 0.05 vs. control stressed cells; # *p* < 0.05 vs. empty liposomes. All samples were different from control cells (without pretreatment or H_2_O_2_ exposure, 100% viability).

The antioxidant activity of *C. siliqua* samples was also investigated as the ability to protect cells from hydrogen-peroxide-induced oxidative stress. An extract concentration of 100 µg/mL was used for each cell pretreatment. Figure 5A displays that the exposure to 2 mM H_2_O_2_ reduced 3T3 viability to 38% compared to the untreated cells and that there was some protection capacity with the pretreatment with the extract solution (approximately 7%), which was enhanced when the extract was formulated in liposomes (approximately 31%). In the case of HaCaT cells, H_2_O_2_ induced a cytotoxic effect similar to that induced in 3T3 cells (41%). The pretreatment with the extract solution had a protective effect, increasing cell viability to 55%. Liposomes showed a lower protection, not significantly different from the solution (Figure 5B). The same trend was found in tumoral A431 keratinocytes (Figure 5C).

## 4. Discussion

*C. siliqua* pods are known for their antioxidant properties that are of value for the amelioration and prevention of many disorders [33,34]. In the literature, there are many studies comparing different extraction methods to prepare an active extract. The solvent concentration, the extraction time, and the extraction temperature are the parameters that most influence the phytochemical profile of the extracts [15,35,36]. Generally, the ethanol–water mixtures have been found to be effective for the antioxidants’ extraction from botanical materials [36]. In this study, a *C. siliqua* pod extract was prepared through sonication and maceration in a 70:30 (*v*/*v*) ethanol–water mixture. DPPH and FRAP tests showed a prominent antioxidant activity of the prepared extract, which may be related to the high content of hydroxybenzoic acids, especially gallic acid and its derivatives [37,38,39]. The total concentration of the phenolic compounds detected in *C. siliqua* pod extract was ~7.56 mg/g of dried extract, with hydroxybenzoic acids accounting for ca. 95% (7.19 mg/g of dried extract). The *C. siliqua* pod extract efficiently neutralized the DPPH radicals. Similarly, the FRAP assay showed the strong reducing power of the extract. These antioxidant abilities were retained after the nanoformulation process. The latter was performed to increase the bioavailability of the extract components and to produce a formulation feasible for topical application. The liposomes were small in size, with spherical and elongated shapes and oligolamellar arrangement. They entrapped key phenolic compounds with high efficiency and were applied safely in different cell cultures. The strong antioxidant capacity highlighted by colorimetric tests did not translate into an equally strong protection from H_2_O_2_-induced oxidative stress in cellular systems. These discrepancies can be related to the differences between chemical methods (DPPH and FRAP assays) and biological systems using living cells. In addition, the cell response was found to depend on the cell line. The morphological and physiological properties of the cells could explain differences in their sensitivity. Accordingly, while keratinocytes are an example of cells representative of the epidermis, fibroblasts are found in the dermal skin layer. For this reason, 3T3 cells are more sensitive than HaCaT or A431 cells to the deleterious effect of hydrogen peroxide. Interestingly, in the case of tumoral cells (A431), the pretreatment with the extract in liposomes was more effective than in the other cell lines. Klenow et al. showed that a treatment with *C. siliqua* extract reduced DNA damage in human colon cells challenged with hydrogen peroxide [40]. No other results were found in the literature about the *C. siliqua* pod extracts’ ability to prevent H_2_O_2_-induced cytotoxicity, but their ability to prevent or reduce oxidation and inflammation-related disorders are known. Ünal et al. highlighted the antioxidant capacity of a carob pod aqueous extract against deltamethrin-induced oxidative stress, a pesticide widely used around the world, in vitro and in vivo in a zebrafish model [41]. Similarly, Çavuşoğlu et al. evaluated the protective property of *C. siliqua* pod extract against toxicity induced by 1,4-dioxane, a common contaminant present in many industrial products [6]. Al-Olayan et al. investigated the ameliorative effects of *C. siliqua* pod aqueous extract on liver fibrosis and oxidative stress in mice infected with *Schistosoma mansoni*, a parasite responsible for an excessive production of reactive oxygen species (ROS) that induces hepatic stress. The treatment of infected mice with the extract increased hepatic GSH contents (the main endogenous antioxidant) and restored the activities and expression levels of the antioxidant enzymes SOD, CAT, GST, GPx, and GR [42]. Alzoubi et al. showed that a methanolic extract of carob pods prevented the impairment of short-term memory induced by chronic stress in rats, probably as a result of a prevented reduction in brain-derived neurotrophic factor levels in the hippocampus [10]. Ben Ayache et al. analyzed the phytochemical profile of aqueous extracts prepared from Tunisian varieties of carob. The study showed that carob extracts may be implicated in several pathways, contributing to antioxidant activity by means of their bioactive components. The extracts exhibit potent radical scavenging properties, resulting in analgesic activity in mice and proapoptotic capacity in different cancer cell lines [8]. Nevertheless, it is important to underline that none of the above studies involved the use of liposomes. This strengthens the potential of our findings and the need for further investigation.

## 5. Conclusions

The nanoformulation of extracts in phospholipid vesicles is one of the most promising strategies to overcome obstacles related to undesirable features of bioactive compounds and to increase their applicability in therapy. Many skin disorders are related to a prooxidants–antioxidants imbalance. Oxidative stress leads to adverse effects on essential cellular components, such as DNA, proteins, and lipids. Antioxidant products can serve as effective strategies for improving these conditions. The results found in this study point to promising perspectives to exploiting the antioxidant properties of *C. siliqua* pod extract for skincare through liposomal delivery systems.

## Figures and Tables

**Table 1:** Composition of the *C. siliqua* pod extract liposomes and empty liposomes.

	P90G	Extract	Water
Liposomes	150 mg	20 mg	1 mL
Empty liposomes	150 mg		1 mL

**Table 2:** Compound identification by (HR) HPLC-ESI-QToF MS/MS in *C. siliqua* extract.

Peak No.	Rt Min	Identity	[M-H]^−^ * m/*z*	Molecular Formula	Δ ppm	MS/MS ^§^ *m*/*z*	References	Confidence Level #
1	2.64	Gallic acid glucoside	331.0677	C_13_H_16_O_10_	0.3297	271.0456(14)/211.0230(13)/169.0132(100)	[5,29]	2
2	3.38	Gallic acid	169.0144	C_7_H_6_O_5_	0.1532	125.0243(100)	[5,29]	1
3	3.72	(iso)butyryl-dihexose	457.1567 [FA]	C_16_H_28_O_12_	−1.1088	411.1508(9)/342.1085(88)/87.046(12)	[29]	3
4	4.15	Gallic acid glucoside	331.06709	C_13_H_16_O_10_	0.3297	271.0443(65)/211.0243(72)/169.0134(100)	[29]	2
5	4.26	Digalloyl glucose	483.0783	C_20_H_20_O_14_	0.4729	331.0661(47)/313.0562(27)/169.0144(100)	[5,29]	2
6	4.93	Monogalloyl dihexoside	493.1201	C_19_H_26_O_15_	0.8063	313.0564(100)/283.0431(72)/169.0133(54)	[5,29,30]	3
7	5.09	Unknown	417.1172	C_17_H_30_O_14_				4
8	5.35	Monogalloyl dihexoside	493.1201	C_19_H_26_O_15_	0.3063	313.0565(100)/271.0438(65)/169.0129(60)	[29]	3
9	5.51	Monogalloyl dihexoside	493.1201	C_19_H_26_O_15_	1.0063	313.0571(100)/283.0454(63)/169.0135(87)	[29]	2
10	5.90	(iso)Btyryl-dihexose	411.1518	C_16_H_28_O_12_	−1.0943	323.0981(28)/179.0558(9)	[29]	3
11	6.43	(iso)Butyryl-hexose-pentose	427.1466 [FA]	C_15_H_26_O_11_	−0.9178	381.1402(4)/125.0239(31)/87.0460(36)	[29]	3
12	6.65	Digalloyl glucose	483.0783	C_20_H_20_O_14_	0.6233	331.0672(26)/313.0565(20)/169.0141(100)	[5,29]	2
13	6.86	(iso)Butyryl-hexose-pentose	427.1466 [FA]	C_15_H_26_O_11_	−0.793	233.0627(8)/87.0450(37)/59.0144(65)	[29]	3
14	7.16	(iso)Butyryl-hexose-pentose	427.1466 [FA]	C_15_H_26_O_11_	−0.9045	149.0448(6)/87.0456(85)/59.0145(56)	[29]	3
15	7.46	(iso)Butyryl-dihexose	457.1567 [FA]	C_16_H_28_O_12_	−1.1092	341.1090(13)/323.0978(11)/87.0463(44)	[29]	3
16	7.86	(iso)Butyryl-dihexose	411.1518	C_16_H_28_O_12_	−1.0699	341.1083(4)/87.046(35)/59.0145(14)	[29]	3
17	7.96	(iso)Butyryl-dihexose	411.1518	C_16_H_28_O_12_	−1.1092	341.1087(6)/87.0457(34)	[29]	3
18	8.45	(iso)Butyryl-dihexose	411.1518	C_16_H_28_O_12_	−1.1092	341.1086(5)/87.0468(25)	[29]	3
19	9.12	Methyl gallic acid	183.0300	C_8_H_8_O_5_	0.1031	168.0064(14)/124.0169(100)	[5]	2
20	9.66	Unknown	443.1923	C_21_H_32_O_10_	−0.6477	89.0260(41)/71.0145(33)/59.0154(49)	[29]	4
21	10.03	Acylated hexose-pentose	417.1144 [FA]	C_16_H_28_O_11_	0.2744	293.0878(29)/101.0614(100)	[18]	3
22	10.17	Acylated hexose-pentose	441.1615 [FA]	C_16_H_28_O_11_	−1.4446	101.0625(66)/71.0144(47)/59.0151(88)	[29]	3
23	10.59	Acylated hexose-pentose	441.1615 [FA]	C_16_H_28_O_11_	−1.4446	101.0613(49)/71.0123(24)/59.015(61)	[29]	3
24	10.77	Digalloyl glucose	483.0783	C_20_H_20_O_14_	−1.22	331.0669(17)/313.0563(17)/169.0135(94)	[5,29]	2
25	11.04	Unknown	261.0879	C_13_H_14_N_2_O_4_	1.224			4
26	11.13	Trigalloyl glucose	635.0886	C_27_H_24_O_18_	0.8457	465.0682(88)/169.0134(28)	[5,29]	2
27	11.47	Acylated hexose-pentose	461.1296 [FA]	C_18_H_24_O_11_	−0.7563	121.0299(100)	[29]	3
28	12.21	Acylated hexose-pentose	461.1296 [FA]	C_18_H_24_O_11_	−1.0071	267.0890(11)/121.0284(100)	[29]	3
29	12.24	Siliquapyranone	595.1309	C_26_H_28_O_16_	0.4416	483.0773(8)/331.0625(11)/169.0124(100)	[5,29]	2
30	12.89	Trigalloyl glucose isomer	635.0888	C_27_H_24_O_18_	−0.5480	465.0675(94)/169.0126(13)	[5,29]	2
31	13.94	Unknown	186.1140	C_9_H_17_NO_3_	−0.2786		[29]	4
32	14.27	Ethyl gallic acid	197.046	C_9_H_10_O_5_	0.453	169.0134(26)/125.0245(100)/124.0153(58)	[18]	3
33	14.93	Trigalloyl glucose isomer	635.0888	C_27_H_24_O_18_	0.9724	465.0681(92)/169.0124(11)	[5,29]	2
34	16.20	Gallic acid derivative	401.1095	C_17_H_22_O_11_	0.5649	169.0138(100)/123.0084(65)/101.0616(50)	[5]	2
35	17.51	Benzoic acid	121.02935	C_7_H_6_O_2_	−0.3030	77.0397(100)	[31]	2
36	18.99	Myricetin deoxyhexoside	463.0876	C_21_H_20_O_12_	−0.5996	317.0272(16)/316.0207(100)	[5,29]	2
37	19.76	Quercetin hexoside	463.0876	C_21_H_20_O_12_	0.1004	301.0359(39)/300.0271(100)	[5,29]	2
38	20.25	*p*-Coumaroyl galloyl hexose	477.10464	C_22_H_22_O_12_	−0.2197	331.0645(21)/169.0138(100)/125.0234(31)	[5]	2
39	20.65	Quercetin pentoside	433.07735	C_20_H_18_O_11_	−1.1749	301.0337(59)/300.0283(100)	[5,29]	2
40	21.11	Gallic acid derivative	507.11438	C_23_H_24_O_13_	−1.2278	235.0621(100)/169.0156(83)	[5]	2
41	21.42	Unknown	435.09317	C_20_H_20_O_11_	−0.9888			4
42	22.01	Quercetin-3-*O*-rhamnoside	447.09389	C_21_H_20_O_11_	0.9150	301.0336(40)\300.0273(100)\271.0248(16)	[5,29]	1
43	24.81	Kaempferol deoxyhexoside	431.09883	C_21_H_20_O_10_	1.7355	285.0404(19)/284.0294(22)/255.0280(31)	[5,29]	2
44	26.23	Tetrahydroxy flavanone	287.05581	C_15_H_12_O_6_	−0.9017	151.0042(100)\135.0459(58)	[5]	2
45	28.73	Kaempferol	285.0441	C_15_H_9_O_6_	0.0384	/	[5,29]	1

* FA: formic acid adduct; ^§^ in parentheses the relative intensity; # according to [28].

**Table 3:** Amounts of phenolic compounds in *C. siliqua* pod extract (mg/g of dried extract (dr); means ± SD, *n* = 3).

Compound	Peak No. ^§^	*C. siliqua* Extract (mg/g dr)	
		**Mean**	**±SD**
**Total Hydroxybenzoic acids**		**7.20**	**0.37**
Gallic acid hexose ^a^	1	1.51	0.00
Gallic acid	2	1.31	0.01
Gallic acid hexose ^a^	4	0.06	0.00
Digalloyl glucose ^a^	5	0.06	0.00
Monogalloyl dihexoside ^a^	6	0.58	0.01
Monogalloyl dihexoside ^a^	8	0.15	0.00
Monogalloyl dihexoside ^a^	9	0.08	0.00
Digalloyl glucose ^a^	12	0.22	0.00
Methyl gallic acid ^a^	19	0.36	0.00
Digalloyl glucose ^a^	24	0.15	0.00
Trigalloyl glucose ^a^	26	0.14	0.00
Siliquapyranone ^a^	29	0.15	0.00
Trigalloyl glucose isomer ^a^	30	0.19	0.00
Ethyl gallic acid ^a^	32	0.29	0.00
Trigalloyl glucose isomer ^a^	33	0.11	0.00
Gallic acid derivative ^a^	34	1.70	0.01
Gallic acid derivative ^a^	40	0.14	0.00
**Total Flavonoids**		**0.36**	**0.02**
Myricetin deoxyhexoside ^b^	36	0.05	0.00
Quercetin hexoside ^c^	37	0.04	0.00
Quercetin pentoside ^c^	39	0.02	0.01
Quercetin-3-*O*-ramnoside	42	0.21	0.00
Kaempferol deoxyhexoside ^d^	43	0.01	0.00
Tetrahydroxy flavanone ^e^	44	0.02	0.00
Kaempferol	45	0.01	0.00
**Total polyphenols**		**7.56**	**0.41**

^a^ Expressed as gallic acid equivalents; ^b^ expressed as myricetin-3-*O*-glucoside equivalents; ^c^ expressed as quercetin-3-*O*-ramnoside equivalents; ^d^ expressed as kaempferol-3-*O*-glucoside equivalents; ^e^ expressed as quercetin equivalents; ^§^ peak number as reported in Table 2.

**Table 4:** Characteristics of *C. siliqua* liposomes and empty liposomes. Each value represents the mean ± SD (*n* > 10).

	Liposomes	Empty Liposomes
Mean diameter (nm ± SD)	* 107 ± 3.8	73 ± 2.0
Polydispersity index (± SD)	0.27 ± 0.01	0.31 ± 0.03
Zeta potential (mV ± SD)	−13 ± 2.8	−18 ± 2.7

* Values statistically different (*p* < 0.01) from empty liposomes.

**Table 5:** Fitting parameters and derived parameters (± estimated error from the fit) for SAXS curves of *C. siliqua* liposomes and empty liposomes. χ_2_: reduced chi squared, *N*: number of correlated layers, *d*: repetition distance, *η*_1_: Caillé parameter, Z_H_: polar head Gaussian center, σ_H_: polar head Gaussian amplitude, and σ_C_: methyl Gaussian amplitude.

	Liposomes	Empty Liposomes
*χ* ^2^	1.75	1.61
*N*	1.26	1.00
*d* (*Å*)	62.16	/
*η*_1_	0.23	/
*Z_H_* (*Å*)	16.05 ± 0.20	15.60 ± 0.20
*σ_H_* (*Å*)	3.44 ± 0.20	4.16 ± 0.20
σ_C_ (*Å*)	5.19 ± 0.50	7.48 ± 0.50

**Table 6:** Entrapment efficiency (EE) of two phenolic compounds (one hydroxybenzoic acid and one flavonoid) identified in *C. siliqua* extract. Data are given as the means ± SD (*n* = 4).

Peak No. ^§^	Compound	EE (% ± SD)
33	Trigalloyl glucose ^a^	97 ± 6.3
42	Quercetin-3-*O*-rhamnoside	91 ± 7.7

^a^ Expressed as gallic acid equivalents. Data are given as means ± standard deviations (*n* = 4). ^§^ Peak number as reported in Table 2.

**Table 7:** Hemolytic activity of *C. siliqua* extract in solution and in liposomes. For a proper comparison, empty liposomes were subjected to the same dilutions used for the extract samples. Data are expressed as % means ± standard deviations (SD); *n* = 2.

	Extract Concentration mg/mL	Hemolytic Activity (% ± SD)
Solution	1	1.7 ± 0.70
Liposomes	1	2.4 ± 2.89
Empty liposomes	/	1.4 ± 1.14
Solution	2	1.6 ± 1.07
Liposomes	2	0.9 ± 1.33
Empty liposomes	/	3.7 ± 1.92

**Table 8:** The DPPH assay results are expressed as AA (%) and TE (μg Trolox equivalents/mL solution) and reported as the means ± SD of at least three separate experiments, each performed in triplicate.

	AA (%)	TE (µg Trolox Equivalents/mL)
Solution	92 ± 4.2	469 ± 14.1
Liposomes	95 ± 1.7	* 486 ± 10.5
Empty liposomes	40 ± 4.1	220 ± 18.2

* Value statistically different (*p* < 0.05) from the extract solution.

**Table 9:** The FRAP assay results are expressed as FE (µg Fe^2+^ equivalents/mL solution) and reported as the means ± SD of at least three separate experiments, each performed in triplicate.

	TE (µg Fe^2+^ Equivalents/mL Solution)
Solution	2139 ± 257
Liposomes	1995 ± 253
Empty liposomes	687 ± 99

## Data Availability

The data presented in this study are available within this article.
